# Pleuromutilins Suppress Hepatocellular Carcinoma Growth via ABCA1 Inhibition-Induced Cholesterol Accumulation

**DOI:** 10.3390/cancers18071054

**Published:** 2026-03-24

**Authors:** Mingshan Zhou, Jie Cao, Junfei Chen, Bohan Zhang, Jiawen Wu, Xiaofeng Lian, Miaoxin Zhu, Peifeng Liu, Min Zhou

**Affiliations:** 1 State Key Laboratory of Systems Medicine for Cancer, Shanghai Cancer Institute, Renji Hospital, Shanghai Jiao Tong University School of Medicine, Shanghai 200032, China; 18111520013@fudan.edu.cn (M.Z.); jf_chen@zju.edu.cn (J.C.); bohan6309@sjtu.edu.cn (B.Z.); wjw-2001@sjtu.edu.cn (J.W.); lianxiaofeng@sjtu.edu.cn (X.L.); zhumiaoxin@renji.com (M.Z.); 2 Department of Liver Surgery, Renji Hospital, School of Medicine, Shanghai Jiao Tong University, 160 Pujian Road, Shanghai 200127, China; caojie@renji.com; 3 Department of Thoracic Surgery, Shanghai Chest Hospital, Shanghai Jiao Tong University School of Medicine, Shanghai 200030, China

**Keywords:** hepatocellular carcinoma, lefamulin, tiamulin, ABCA1, cholesterol metabolism, tumor immunity

## Abstract

Bcakground: Hepatocellular carcinoma (HCC) is a prevalent malignancy with limited therapeutic options. Drug repurposing offers an attractive strategy to accelerate anticancer discovery. The pleuromutilin class of antibiotics, including the human-approved agent lefamulin and the veterinary drug tiamulin, has shown preliminary anticancer potential, but its efficacy and mechanism in HCC remain unexplored. Methods: The anti-tumor effects of lefamulin and tiamulin were evaluated in HCC cell lines, patient-derived organoids, and a C57BL/6 mouse subcutaneous tumor model. Safety was assessed in a human normal hepatocyte cell line and by histopathological examination of major organs in treated mice. Mechanistic investigations were performed using RNA-sequencing, RT-qPCR, immunohistochemistry (IHC), filipin staining, pharmacological rescue assays, and shRNA-mediated gene silencing. Results: In this study, we found that both lefamulin and tiamulin markedly inhibited HCC cell proliferation in vitro and significantly suppressed tumor growth in vivo (lefamulin vs. control, *p* = 0.014; tiamulin vs. control, *p* = 0.021), without causing significant toxicity. RNA-sequencing analysis revealed consistent downregulation of the cholesterol transporter *Abca1* (ATP-binding cassette transporter A1) and alterations in cell adhesion molecule pathways. Functional studies confirmed that treatment reduced ABCA1 protein levels, leading to intracellular cholesterol accumulation and aberrant distribution. Furthermore, treated tumors exhibited a significant increase in CD8^+^ T-cell infiltration, with CD4^+^ T cells and macrophage infiltration remained unchanged, indicating a specific modulation of the tumor immune microenvironment. Conclusions: These findings suggest that lefamulin and tiamulin are promising therapeutic candidates for HCC.

## Simple Summary

This study investigates whether pleuromutilin antibiotics, traditionally used to treat bacterial infections, also possess anti-tumor activity against hepatocellular carcinoma (HCC). HCC is a highly lethal cancer with limited therapeutic options. We found that pleuromutilins significantly inhibited HCC tumor growth in both in vitro and in vivo models by downregulating ATP-binding cassette transporter A1 (ABCA1), a key mediator of cellular cholesterol efflux. Suppression of ABCA1 led to intracellular cholesterol accumulation in liver cancer cells, triggering cellular stress and subsequent tumor cell death. In addition, pleuromutilin treatment altered cell adhesion–related signaling pathways and markedly increased CD8^+^ T cell infiltration, suggesting modulation of the tumor immune microenvironment. Collectively, these findings indicate that pleuromutilins could be repurposed as cost-effective anticancer agents, potentially accelerating drug development and expanding treatment options for patients with HCC.

## 1. Introduction

Hepatocellular carcinoma (HCC) is one of the most prevalent and lethal malignancies worldwide. In China, although its incidence has declined in recent years, HCC remains the fourth most commonly diagnosed cancer and the second leading cause of cancer-related mortality [1]. Although the development of new chemotherapeutic agents and immunotherapies has improved patient outcomes [2,3,4,5], the prognosis of HCC remains poor, with a five-year survival rate lower than that of many other malignancies [1]. Therefore, identifying new therapeutic targets and effective treatment strategies remains an urgent clinical need.

Pleuromutilin antibiotics, which inhibit bacterial protein synthesis via the 50S ribosomal subunit, have recently attracted attention for their potential antitumor effects beyond antimicrobial activity. Lefamulin, approved for community-acquired bacterial pneumonia [6], has recently been shown to overcome sorafenib resistance in HCC [7]. Similarly, the related drug tiamulin has been shown to reverse drug resistance in various tumor cell lines [8] and inhibit breast cancer growth [9]. While these findings highlight the potential of pleuromutilins as anticancer agents, their mechanisms of action in HCC remain incompletely understood.

In this study, we demonstrated that pleuromutilin antibiotics lefamulin and tiamulin inhibit HCC proliferation across in vitro (cell lines and patient-derived organoids) and in vivo (subcutaneous mouse tumors) models. Mechanistically, RNA-sequencing (RNA-seq) analysis revealed downregulation of *Abca1* and altered cell adhesion pathways. These changes were associated with functional outcomes: intracellular cholesterol accumulation in HCC cells and enhanced CD8^+^ T cell infiltration in tumors. Both compounds also exhibited preferential cytotoxicity toward malignant hepatocytes compared with normal hepatocytes, suggesting a potentially favorable therapeutic window. Collectively, these findings indicate that pleuromutilins suppress tumor growth and may also act as immunomodulatory agents, thereby providing a rationale for their integration into combination immunotherapy strategies.

## 2. Materials and Methods

### 2.1. Cell Lines and Culture

Hepa1-6, Hep3B2.1-7 and THLE-2 cell lines were obtained from the American Type Culture Collection (ATCC), and HuH7 cells were obtained from the RIKEN Cell Bank. Hepa1-6, Hep3B2.1-7 and HuH7 were cultured in DMEM medium supplemented with 10% fetal bovine serum and 1% penicillin-streptomycin. THLE-2 cells were cultured in Advanced DMEM/F12 medium supplemented with of 10% fetal bovine serum, 5 ng/mL EGF and 70 ng/mL phosphorylethanolamine. All cells were maintained at 37 °C with 5% CO_2_.

### 2.2. Patient-Derived Organoids (PDOs) and Culture

PDOs were established from resected HCC tissues collected at Renji Hospital in 2025. The study protocol was approved by the Ethics Committee of Renji Hospital, Shanghai Jiao Tong University. Written informed consent for research use of surplus tissue and PDO generation was obtained from all patients prior to surgery. Tumor fragments were de-identified, transported on ice, and processed for organoid culture within 3 h of resection. Tumor tissues were minced and digested with (0.25 mg/mL of collagenase I (0.25 mg/mL) and collagenase IV (1.5 mg/mL) at 37 °C 10–20 min. Cells were washed, resuspended in Matrigel (3.3 mg/mL), and seeded into 96-well U-bottom plates (1 × 10^4^ cells, 10 μL per well). PDOs were cultured in Advanced DMEM/F12 medium supplemented with R-spondin 1, Noggin, Wnt-3a, EGF, HEPES, Glutamax, N2, B27, n-Acetylcysteine, Normocin, Penicillin-Streptomycin, Niacinamide, Gastrin, Prostaglandin E2, A83-01, SB202190, FGF, Forskolin, TGFa, Dexamethasone, HGF, and Minocycline hydrochloride [10,11,12,13].

### 2.3. Drug Sensitivity Assay

HCC cells and PDOs were plated in 96-well plates at densities of 2500 cells and 1000 organoids per well, respectively. Cells were treated with lefamulin or tiamulin (TargetMol) at indicated concentrations. Growth was monitored using a Celigo Image Cytometer (Nexcelom, Boston, MA, USA), with cell confluence analyzed by the instrument software and organoid counts quantified using ImageJ (V1.8.0.112 ).

### 2.4. Animal Studies

Animal experiments were approved by the Ethics Committee of Renji Hospital, Shanghai Jiao Tong University. Female C57BL/6 mice (6 weeks old) were implanted subcutaneously with 1 × 10^7^ Hepa1-6 cells in 150 µL PBS. When tumors reached ~150 mm^3^, mice were randomized to receive daily intraperitoneal injections of lefamulin (37 mg/kg), tiamulin (37 mg/kg), or saline (control). Tumor volumes were measured every two days using digital calipers and calculated with the formula: volume = (length × width^2^)/2. Mice were euthanized when any tumor reached 1000 mm^3^. Tumors were excised, weighed, and processed for downstream analyses.

### 2.5. RNA-Sequencing Analysis

Total RNA was extracted from Hepa1-6 cells treated with 37 µg/mL lefamulin, 37 µg/mL tiamulin, or vehicle control using TRIzol reagent (Thermo Fisher Scientific, Waltham, MA, USA). RNA-seq analysis was performed by Novogene (Shanghai, China). Differentially expressed genes (DEGs) were identified using thresholds of |log_2_(fold change)| > 1 and adjusted *p*-value < 0.05. KEGG and GO enrichment analyses were performed on DEGs. Raw RNA-seq data have been deposited in the NCBI Gene Expression Omnibus (GEO) under accession number GSE315390.

### 2.6. Hematoxylin and Eosin (H&E) Staining

Major organs (heart, liver, spleen, lung, and kidney) were fixed, paraffin embedded sectioned at 5 μm, and stained with hematoxylin and eosin. Whole-slide images were acquired using a NanoZoomer S360 (Hamamatsu Photonics, Hamamatsu, Japan) digital slide scanner.

### 2.7. Immunohistochemical (IHC)

Tumor samples were fixed, paraffin-embedded, and sectioned at 5 µm. Sections were deparaffinized in xylene and rehydrated through a graded ethanol series, and endogenous peroxidase activity was quenched by incubation in 3% hydrogen peroxide in methanol. Heat-induced antigen retrieval was performed by immersing slides in boiling retrieval buffer for 10 min, followed by cooling to room temperature. Non-specific binding was blocked with 5% goat serum for 30 min at room temperature. Sections were then incubated overnight at 4 °C with primary antibodies against ABCA1 (Abcam, Cambridge, UK, ab18180), CD8 (Abcam, Cambridge, UK, ab237709), CD4 (Abcam, Cambridge, UK, ab183685), and F4/80 (Abcam, Cambridge, UK, ab111101). The following day, slides were washed three times with PBS and incubated with an HRP-conjugated secondary antibody (Kangchen, Shanghai, China, KC-MM-035) for 1 h at 37 °C. Chromogenic development was performed using DAB (ShareBio, Shanghai, China) for 10–20 min, followed by counterstaining with hematoxylin for 2 min. Sections were rinsed under running tap water for 20 min, dehydrated through a graded ethanol series and xylene, and mounted with neutral resin. Whole-slide images were acquired using a NanoZoomer S360 digital slide scanner. Positive cells were quantified using ImageJ software (V1.8.0.112).

### 2.8. Filipin Staining

A filipin (Beyotime, Shanghai, China) stock solution was prepared at 50 mg/mL in dimethyl sulfoxide (DMSO, Sigma-Aldrich, St. Louis, MO, USA) and diluted in PBS (1:200, *v*/*v*) before use. Hepa1-6, Hep3B2.1-7, and HuH7 cells were treated with 37 µg/mL lefamulin or tiamulin for 72 h. Cells were then fixed with 4% paraformaldehyde for 10 min at room temperature. Following fixation, cells were stained with the diluted filipin solution for 15 min in the dark, washed three times with PBS, and imaged using a Leica confocal microscope (Leica Microsystems, Wetzlar, Germany). Mean fluorescence intensity of filipin staining was quantified with ImageJ software (V1.8.0.112).

### 2.9. Pharmacological Intervention

Simvastatin was activated by incubation in 10 mM NaOH at 50 °C for 2 h, followed by neutralization with HCl before use. HCC cells were seeded in 96-well plates at a density of 2500 cells per well. Cells were treated with 37 μg/mL lefamulin, 37 μg/mL lefamulin plus 3 μM simvastatin (TargetMol, Boston, MA, USA), 37 μg/mL tiamulin, 37 μg/mL tiamulin plus 3 μM simvastatin, or 5 μg/mL U18666A (TargetMol). Cell growth was monitored daily using a Celigo Image Cytometer (Nexcelom, Boston, MA, USA), and cell confluence was quantified using the instrument’s integrated analysis software.

### 2.10. shRNA Interference

HuH7 cells were seeded in 96-well plates at a density of 2500 cells per well and transduced with viral vectors expressing ABCA1 shRNA (target sequences: GCCTCGTGAAGTATGGAGAAA and CCTCCGAGTCAAGAAGTTAAT). Cell growth was monitored daily using a Celigo Image Cytometer, and cell confluence was quantified using the instrument’s integrated analysis software. Viral particles were produced by transfecting HEK293T cells with the corresponding plasmids.

### 2.11. Statistical Analysis

Data are presented as means ± SEM. Statistical analysis was performed using GraphPad Prism 10.6.1. Comparisons between two groups were made using a two-tailed Student’s *t*-test or a non-parametric test, as appropriate. Multiple group comparisons were analyzed by one-way ANOVA or two-way ANOVA. The half-maximal inhibitory concentration (IC_50_) was calculated using nonlinear regression. Statistical significance is indicated as follows: * *p* < 0.05, ** *p* < 0.01, *** *p* < 0.001, and **** *p* < 0.0001. Non-significant differences are denoted as “n.s.”

## 3. Results

### 3.1. Lefamulin and Tiamulin Inhibit HCC Cell Growth In Vitro

**Figure 1:**
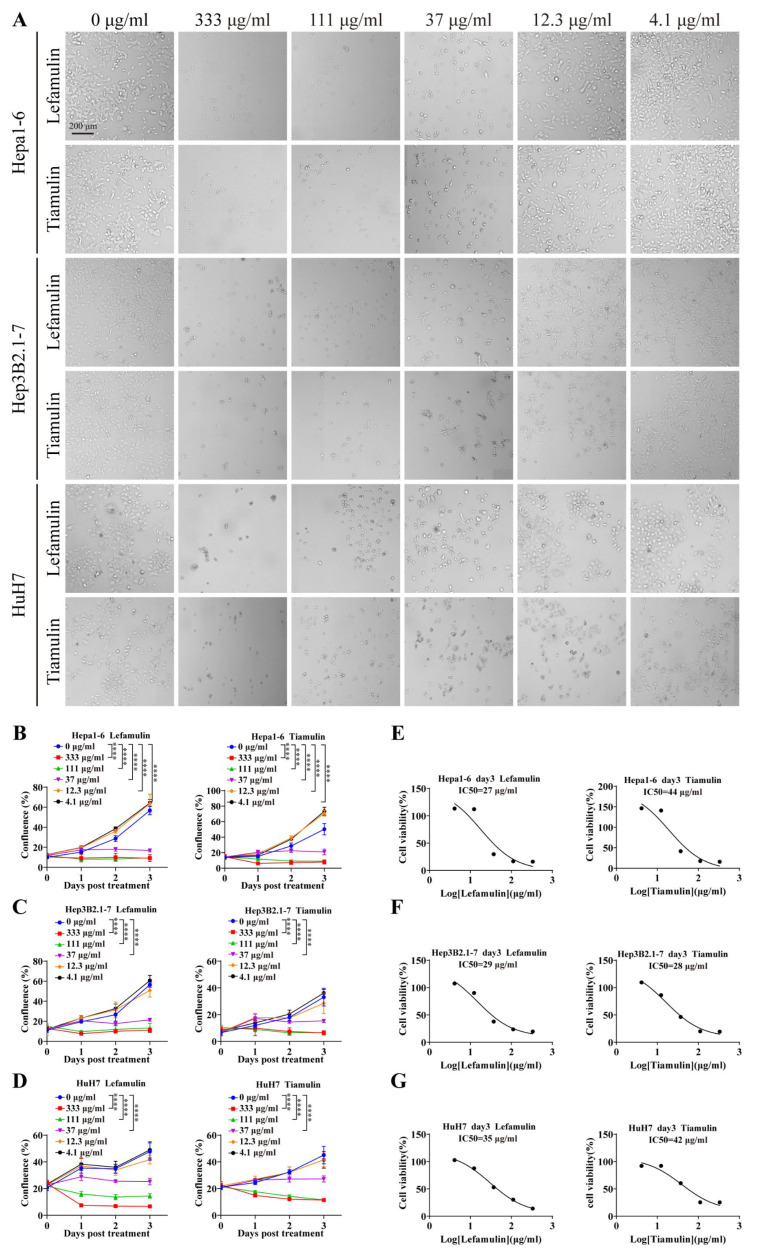
Lefamulin and tiamulin inhibit HCC cell growth in vitro. (**A**) Representative images of HCC cells (Hepa1-6, Hep3B2.1-7 and HuH7) treated with the indicated concentrations of lefamulin or tiamulin for 3 days. Scale bar, 200 µm. (**B**–**D**) Cell proliferation curves of Hepa1-6 (**B**), Hep3B2.1-7 (**C**), and HuH7 (**D**) during treatment with increasing concentrations of lefamulin or tiamulin (*n* = 6). (**E**–**G**) Cell viability was measured after 3 days of treatment with increasing concentrations of lefamulin or tiamulin for Hepa1-6 (**E**), Hep3B2.1-7 (**F**), and HuH7 (**G**) (*n* = 6). Data are expressed as mean ± SEM. Statistical significance was determined by two-way ANOVA; **** *p* < 0.0001.

Lefamulin, an FDA-approved pleuromutilin antibiotic for the treatment of community-acquired bacterial pneumonia [6], was evaluated for its inhibitory effect on hepatocellular carcinoma (HCC) in vitro. Mouse Hepa1-6 cells and human Hep3B2.1-7 and HuH7 were treated with increasing concentrations of lefamulin or its pleuromutilin analogue tiamulin for 3 days (Figure 1A). Both compounds significantly inhibited the proliferation of all tested cell lines at concentrations ≥ 37 μg/mL (Figure 1B–D). The calculated IC_50_ values for lefamulin were 27 μg/mL (Hepa1-6), 29 μg/mL (Hep3B2.1-7), and 35 μg/mL (HuH7), whereas the corresponding IC_50_ values for tiamulin were 44 μg/mL, 28 μg/mL, and 42 μg/mL, respectively (Figure 1E–G). Interestingly, at lower concentrations (12.3 μg/mL and 4.1 μg/mL), both compounds increased the proliferation of Hepa1-6 cells (Figure 1B), with tiamulin showing a more pronounced effect, indicating that their antitumor activity is dose-dependent.

**Figure 2:**
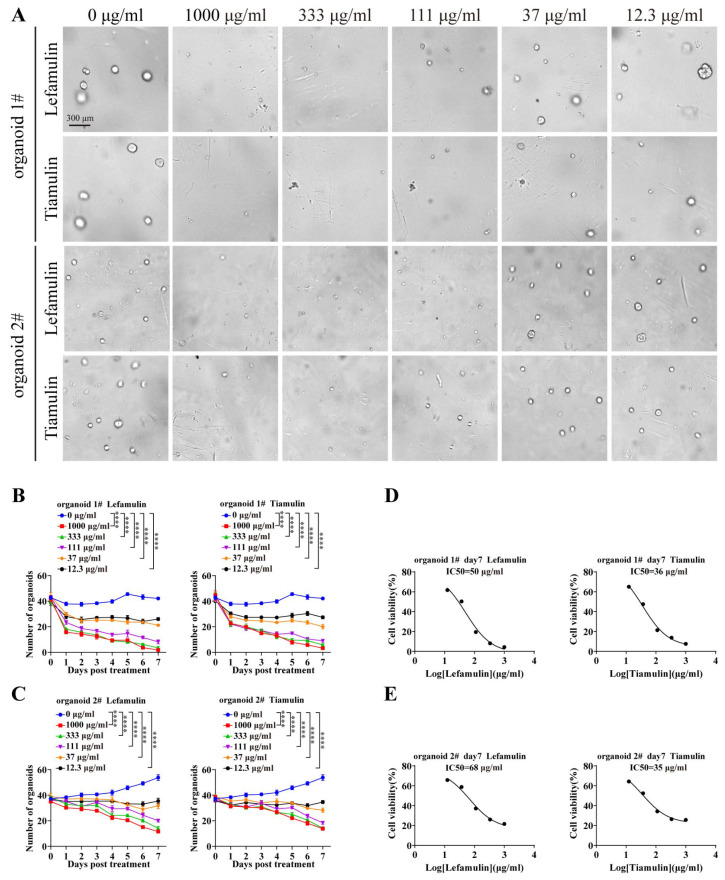
Lefamulin and tiamulin inhibit the growth of HCC organoids. (**A**) Representative images of two patient-derived HCC organoids (Organoid 1# and Organoid 2#) treated with the indicated concentrations of lefamulin or tiamulin for 7 days. Scale bar, 300 µm. (**B**,**C**) Organoid growth curves during treatment with increasing concentrations of lefamulin or tiamulin in Organoid 1# (**B**) and Organoid 2# (**C**). (**D**,**E**) Cell viability of Organoid 1# (**D**) and Organoid 2# (**E**) following treatment with lefamulin or tiamulin. Data are expressed as mean ± SEM. *n* = 5. Statistical significance was assessed by two-way ANOVA; **** *p* < 0.0001.

To further evaluate the inhibitory effects of lefamulin and tiamulin, HCC patient-derived organoids (PDO 1# and 2#) were treated with both compounds for 7 days (Figure 2A). Consistent with the results observed in cell lines, organoid growth was significantly suppressed at concentrations ≥ 37 μg/mL (Figure 2B,C). The IC_50_ values for lefamulin were 50 μg/mL and 68 μg/mL for PDO 1# and PDO 2#, respectively, whereas the corresponding IC_50_ values for tiamulin were 36 μg/mL and 35 μg/mL, respectively (Figure 2D,E). These findings further support the antitumor efficacy of pleuromutilins in HCC in vitro.

### 3.2. Lefamulin and Tiamulin Suppress HCC Tumor Growth In Vivo

**Figure 3:**
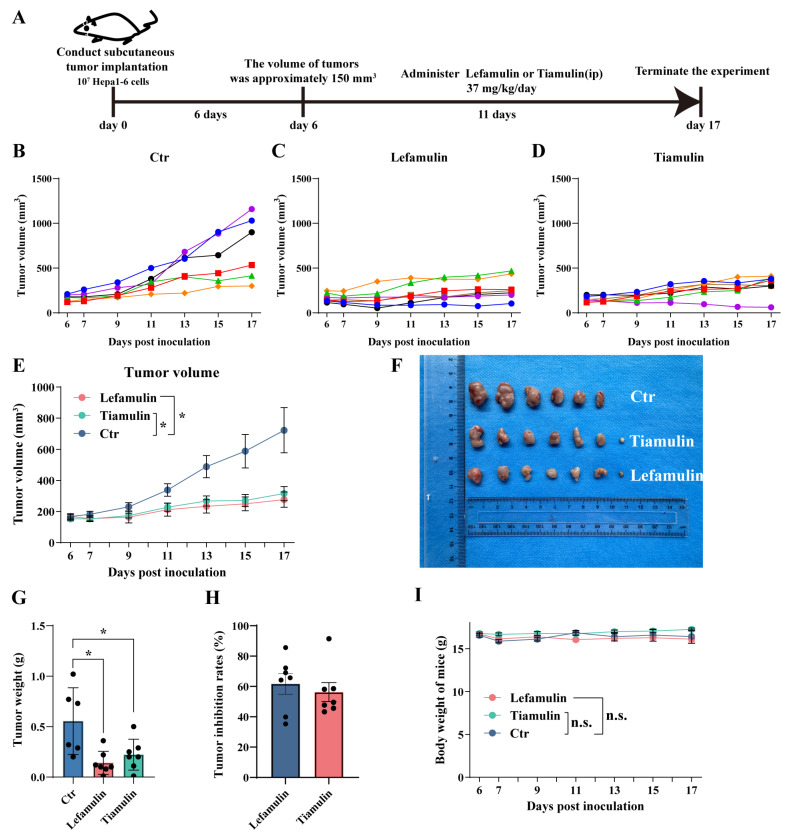
Lefamulin and tiamulin suppress HCC tumor in vivo. (**A**) C57BL/6 mice bearing subcutaneous Hepa1-6 tumors were treated with lefamulin or tiamulin (37 mg/kg/day, intraperitoneally). Group sizes were *n* = 6 (control), *n* = 7 (lefamulin), and *n* = 7 (tiamulin). (**B**–**D**) Individual tumor growth curves (each curve represents an individual mouse) in the control group (**B**, *n* = 6), lefamulin-treated group ((**C**), *n* = 7) and tiamulin-treated group (**D**, *n* = 7) are shown. (**E**) Mean tumor growth curves. (**F**) Representative tumors. (**G**) Tumors weights on day 17. (**H**) Tumor inhibition rates on day 17. (**I**) Changes in body weight. Data are presented as mean ± SEM. Statistical analysis was performed using one-way ANOVA or two-way ANOVA; n.s. not significant, * *p* < 0.05.

To further evaluate the inhibitory effects of lefamulin and tiamulin on HCC, a subcutaneous xenograft model was established by implanting Hepa1-6 cells (1 × 10^7^ /mouse) into C57BL/6 mice (Figure 3A). Treatment with lefamulin or tiamulin (37 mg/kg/day, intraperitoneally) commenced when tumors reached ~150 mm^3^. Both drugs significantly suppressed tumor growth compared with the control group (Figure 3B–F, lefamulin vs. control, *p* = 0.014; tiamulin vs. control, *p* = 0.021). After 11 days of treatment, mean tumor volumes were 277 ± 120 mm^3^ (lefamulin) and 316 ± 111 mm^3^ (tiamulin), compared with 723 ± 323 mm^3^ in controls (Figure 3B–F). At the study endpoint (day 17), mean tumor weights were 0.141 ± 0.106 g (lefamulin) and 0.223 ± 0.142 g (tiamulin), significantly lower than 0.555 ± 0.301 g in controls (Figure 3G, lefamulin vs. control, *p* = 0.0128; tiamulin vs. control, *p* = 0.0379). Correspondingly, tumor inhibition rates were 61.70 ± 16.62% for lefamulin and 56.32 ± 15.32% for tiamulin (Figure 3H). Importantly, no significant changes in body weight were observed during treatment (Figure 3I), suggesting limited systemic toxicity.

### 3.3. Safety Profile of Lefamulin and Tiamulin in Normal Cells and Tissues

**Figure 4:**
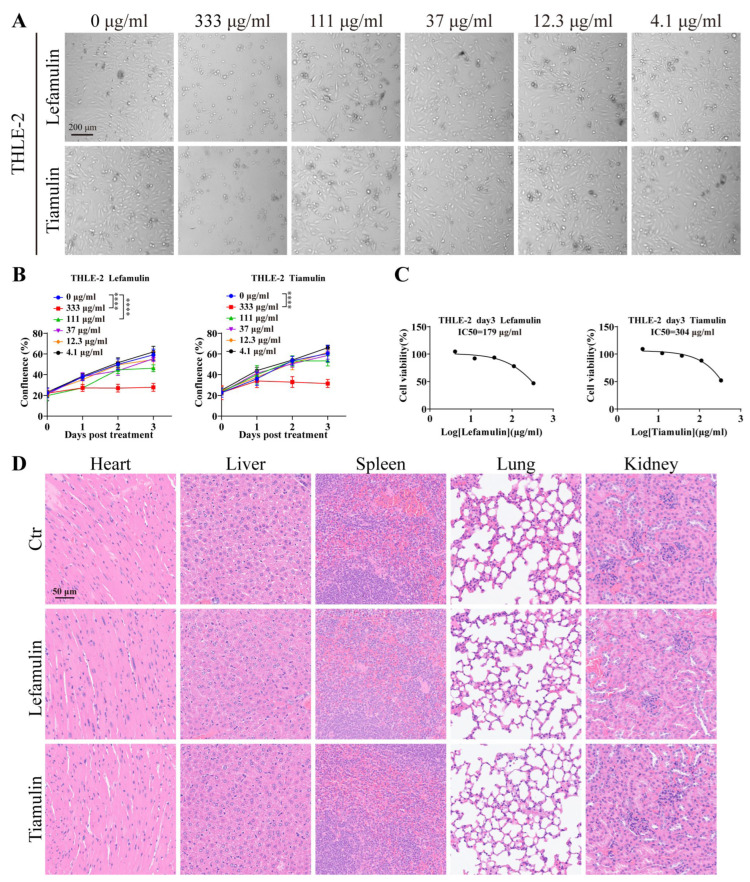
Safety evaluation of lefamulin and tiamulin in normal hepatocytes and major organs. (**A**) Representative images of the normal hepatocyte line THLE-2 following a 3-day treatment with lefamulin or tiamulin at the indicated concentrations. Scale bar, 200 µm. (**B**) The proliferation curves of THLE-2 cells were plotted during treatment with increasing concentrations of lefamulin or tiamulin. (**C**) Cell viability of THLE-2 cells was measured after 3 days of treatment with increasing concentrations of lefamulin or tiamulin (*n* = 6). (**D**) Representative H&E-stained sections of the heart, liver, spleen, lung, and kidney from mice treated with 37 mg/kg lefamulin or tiamulin for 11 days. Scale bar, 50 µm. Data are presented as mean ± SEM. Statistical significance was determined by two-way ANOVA (**** *p* < 0.0001).

Given that the IC_50_ values for lefamulin and tiamulin in tumor models are higher than those typically associated with antibacterial therapeutic exposure [6], a reevaluation of their safety was warranted. The human hepatocyte line THLE-2 was treated with increasing concentrations of lefamulin or tiamulin for 3 days (Figure 4A). Both compounds were well tolerated in THLE-2 cells, with IC_50_ values of 179 μg/mL (lefamulin) and 304 μg/mL (tiamulin, Figure 4B,C). H&E staining of major organs (heart, liver, spleen, lung, and kidney) from treated mice revealed no evident histopathological abnormalities (Figure 4D), supporting a favorable therapeutic window.

### 3.4. Lefamulin and Tiamulin Alter Abca1 and Cell Adhesion Molecule Expression

**Figure 5:**
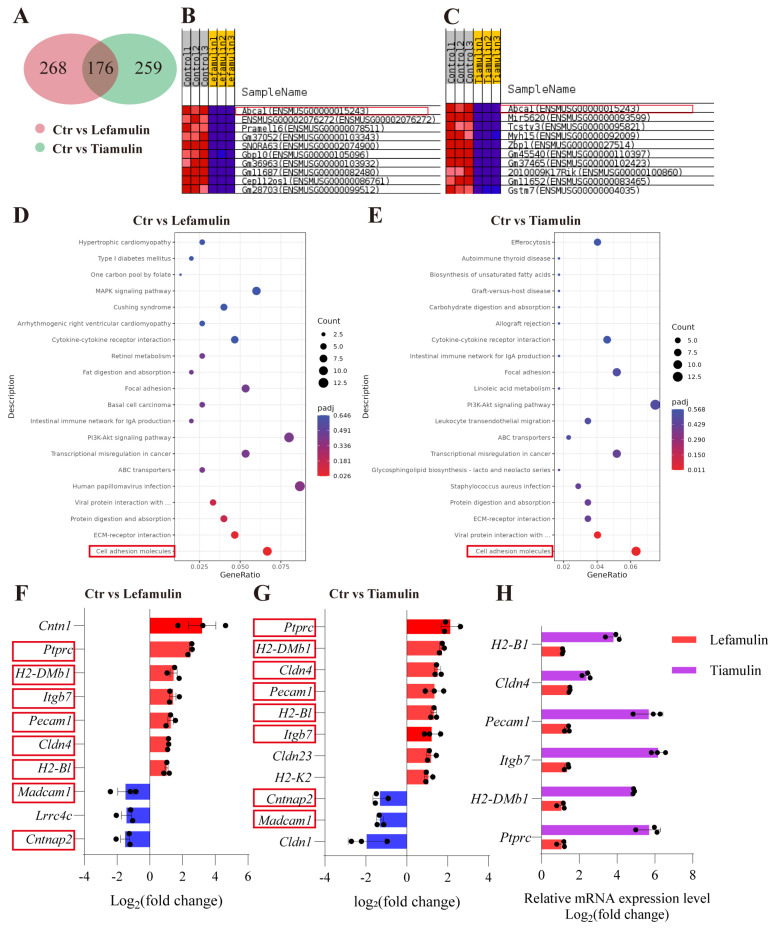
Lefamulin and tiamulin modulate *Abca1* expression and cell adhesion–related pathways. (**A**) Venn diagram illustrating the overlap of differentially expressed genes (DEGs) following lefamulin or tiamulin treatment. (**B**,**C**) Gene Set Enrichment Analysis (GSEA) identified *Abca1* as a prominently downregulated gene in both lefamulin- (**B**) and tiamulin-treated (**C**) cells. (**D**,**E**) KEGG enrichment analysis showed significant alterations in cell adhesion-related signaling pathways after treatment with lefamulin (**D**) or tiamulin (**E**). (**F**,**G**) Differentially expressed genes within cell adhesion molecule signaling pathways after lefamulin (**F**) or tiamulin (**G**) treatment. Genes consistently altered by both treatments are highlighted with red boxes. (**H**) RT-qPCR validation of the differentially expressed genes within cell adhesion molecule signaling pathways.

To investigate the mechanism by which lefamulin and tiamulin inhibit HCC, RNA-Seq analysis was performed on Hepa1-6 cells treated with either compound. Genes with |log_2_(fold change)| > 1 and adjusted *p* value < 0.05 in both treatment groups compared with controls were defined as significantly differentially expressed. The results revealed 176 consistently altered genes (89 upregulated, 87 downregulated) upon treatment with both drugs (Figure 5A). Gene Set Enrichment Analysis (GSEA) identified *Abca1*, a critical regulator of cellular cholesterol efflux [14], as one of the most prominently downregulated genes (Figure 5B,C). KEGG pathway analysis further revealed significant alterations of cell adhesion-related signaling pathways (Figure 5D,E). Specifically, several immune-associated genes were upregulated, including *Ptprc* (protein tyrosine phosphatase receptor type C), *H2-DMb1* (histocompatibility 2, class II, locus Mb1), *Itgb7* (integrin subunit beta 7), *Pecam1* (platelet endothelial cell adhesion molecule 1), *Cldn4* (claudin 4), and *H2-Bl* (histocompatibility 2, blastocyst). In contrast, *Madcam1* (mucosal vascular addressin cell adhesion molecule 1) and *Cntnap2* (contactin-associated protein-like 2) were downregulated (Figure 5F,G). Notably, these genes are involved in key immunological processes. *Ptprc* regulates T-cell activation [15]; *H2-DMb1* and *H2-Bl* participate in antigen presentation [16]; *Itgb7*, *Pecam1* and *Madcam1* contribute to lymphocyte trafficking and recruitment [17,18]; *Cldn4* has been implicated in modulating immune cell infiltration [19]; and *Cntnap2* has been linked to immune regulation functions [20]. Consistent with the transcriptomic findings, RT-qPCR validation confirmed the upregulation of *Ptprc*, *H2-DMb1*, *Itgb7*, *Pecam1*, *Cldn4* and *H2-B1* (Figure 5H, primer sequences listed in Table S1), supporting the notion that lefamulin and tiamulin may reshape the tumor immune microenvironment.

### 3.5. Lefamulin and Tiamulin Reduce ABCA1 Expression and Promote Intracellular Cholesterol Accumulation

**Figure 6:**
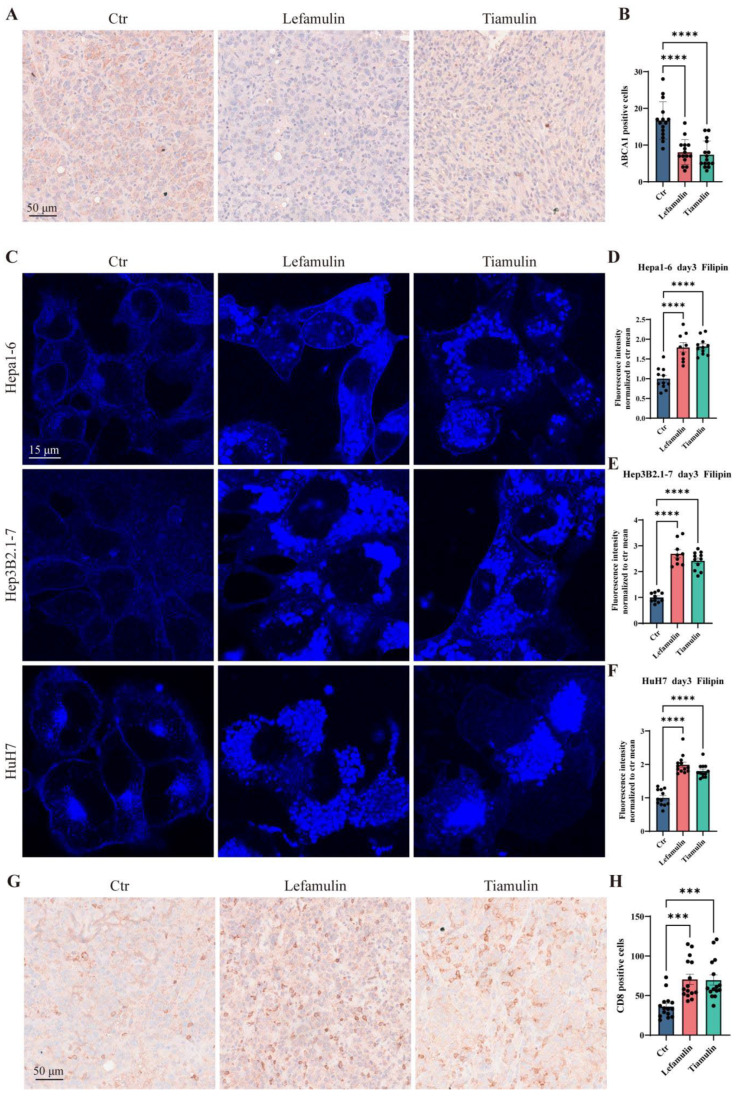
Lefamulin and tiamulin reduce ABCA1 expression, promote intracellular cholesterol accumulation and enhance CD8^+^ T-cell infiltration. (**A**) Representative immunohistochemical images of ABCA1 in tumors of mice treated with 37 mg/kg of lefamulin or tiamulin for 11 days. Scale bar, 50 µm. (**B**) Quantification of ABCA1 expression (*n* = 15). (**C**–**F**) Representative images of filipin staining ((**C**), Scale bar, 15 µm) and quantification of mean fluorescence intensity in Hepa1-6 (**D**), Hep3B2.1-7 (**E**), and HuH7 cells (**F**) treated with 37 µg/mL lefamulin or tiamulin for 3 days. (**G**) Representative immunohistochemical images of CD8 in tumors of mice treated with 37 mg/kg of lefamulin or tiamulin for 11 days. Scale bar, 50 µm. (**H**) Quantification of CD8 positive cells. Data are presented as mean ± SEM. Statistical significance was determined by one-way ANOVA, *** *p* < 0.001, **** *p* < 0.0001.

To validate the downregulation of *Abca1* observed in RNA-Seq analysis, immunohistochemical (IHC) staining for ABCA1 protein was performed on subcutaneous tumor tissues. Quantitative analysis showed a significant reduction in ABCA1-positive cells in tumors from drug-treated mice (lefamulin: 7.40 ± 3.56; tiamulin: 8.07 ± 3.34), versus controls (16.73 ± 4.93, Figure 6A,B). Because ABCA1 plays a critical role in mediating cholesterol efflux, its suppression is expected to result in intracellular cholesterol accumulation [21]. To assess this, we treated Hepa1-6, Hep3B2.1-7, and HuH7 cells with 37 µg/mL of lefamulin or tiamulin for three days and performed filipin staining. In control cells, filipin signal was localized primarily at the plasma membrane and perinuclear region (Figure 6C). In contrast, treatment with either drug induced marked intracellular cholesterol accumulation characterized by increased cytoplasmic and nuclear filipin signals (Figure 6C). Quantitative analysis demonstrated a significant increase in mean fluorescence intensity following treatment. Lefamulin increased filipin fluorescence to 1.79-, 2.70-, and 1.99-fold of control levels in Hepa1-6, Hep3B2.1-7, and HuH7 cells, respectively (Figure 6D–F). Similarly, tiamulin elevated fluorescence to 1.82-, 2.42-, and 1.80-fold of control levels (Figure 6D–F). Collectively, these results demonstrated that lefamulin and tiamulin downregulate ABCA1 expression, leading to intracellular cholesterol accumulation and aberrant cholesterol distribution.

### 3.6. Lefamulin and Tiamulin Enhance CD8^+^ T Cell Infiltration

Cell adhesion molecules are transmembrane glycoproteins that play critical roles in immune cell trafficking and tumor–immune interactions [22]. Their function is closely linked to lipid rafts, cholesterol- and sphingomyelin-enriched membrane microdomains that facilitate the recruitment and signaling of adhesion molecules [23]. We therefore hypothesized that drug-induced disruption of cholesterol homeostasis could alter adhesion molecule activity via lipid raft remodeling, thereby reshaping the tumor immune microenvironment. To test this, we performed immunohistochemistry on subcutaneous tumors to quantify infiltrating CD8^+^ T cells, CD4^+^ T cells, and macrophages. Treatment with lefamulin or tiamulin significantly increased the number of CD8^+^ T cells per field (70.53 ± 20.41 and 69.73 ± 24.39, respectively) compared with controls (36.20 ± 14.51, Figure 6G,H). In contrast, no significant differences were observed in CD4^+^ T cell and macrophage infiltration (Figure S1), suggesting a selective enhancement of cytotoxic T-cell recruitment.

### 3.7. Cholesterol Accumulation Suppresses Cell Proliferation and Alters the Expression of Cell Adhesion Molecules

**Figure 7:**
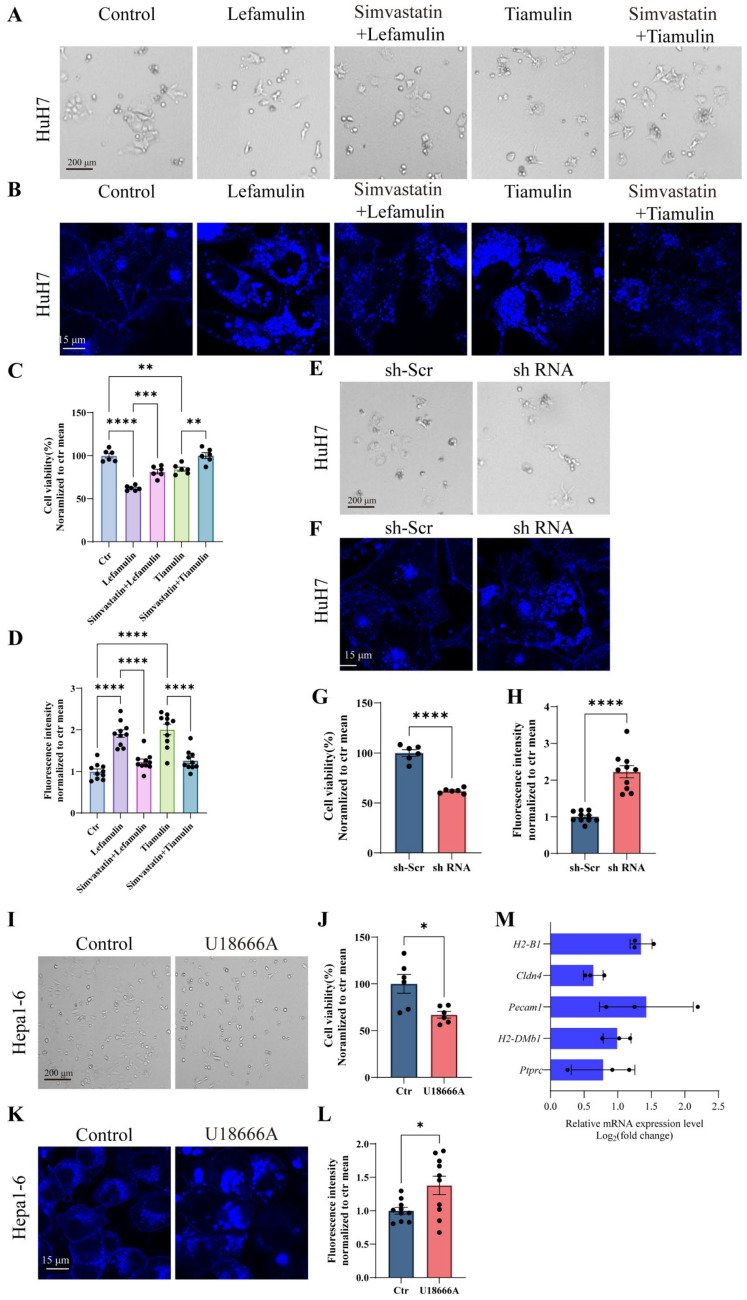
Cholesterol accumulation suppresses cell proliferation and alters the expression of cell adhesion molecules. (**A**,**B**) Representative bright-field images ((**A**); Scale bar, 200 μm) and filipin staining images ((**B**); scale bar, 15 μm) of HuH7 cells treated for 2 days with lefamulin (37 μg/mL), lefamulin plus simvastatin (3 μM), tiamulin (37 μg/mL), or tiamulin plus simvastatin (3 μM). Scale bar, 200 μm. (**C**) Cell viability of HuH7 cells following 2 days of treatment with lefamulin or tiamulin in the presence or absence of simvastatin. (**D**) Quantification of mean filipin fluorescence intensity after the indicated treatments. (**E**,**F**) Representative bright-field images ((**E**); Scale bar, 200 μm) and filipin staining images ((**F**); scale bar, 15 μm) of HuH7 cells transduced with ABCA1 shRNA or scramble control (sh-Scr). (**G**) Cell viability of HuH7 cells following ABCA1 knockdown. (**H**) Quantification of intracellular cholesterol levels based on filipin fluorescence. (**I**) Representative images of Hepa1-6 cells treated with U18666A (5 μg/mL) for 24 h (Scale bar, 200 μm). (**J**) Cell viability of Hepa1-6 cells after U18666A treatment. (**K**,**L**) Representative filipin staining images ((**K**); scale bar, 15 μm) and quantification of mean fluorescence intensity (**L**) in Hepa1-6 cells treated with U18666A. (**M**) RT-qPCR validation of adhesion-related gene expression in Hepa1-6 cells treated with U18666A. Data are presented as mean ± SEM. Statistical significance was determined using one-way ANOVA or Student’s *t*-test; n.s., not significant; * *p* < 0.05, ** *p* < 0.01, *** *p* < 0.001, and **** *p* < 0.0001.

To determine whether cholesterol accumulation contributes to tumor cell inhibition, cholesterol biosynthesis was blocked using 3 μM simvastatin in HuH7 cells. Simvastatin treatment significantly attenuated lefamulin- or tiamulin-induced inhibition of cell proliferation and reduced intracellular cholesterol accumulation, although the abnormal cholesterol distribution persisted (Figure 7A–D). To further validate the role of ABCA1, we knocked down ABCA1 expression in HuH7 cells using shRNA. ABCA1 silencing resulted in reduced cell proliferation and induced cholesterol accumulation with aberrant intracellular distribution (Figure 7E–H), thereby recapitulating key phenotypes observed following pleuromutilin treatment. Next, we investigated whether cholesterol accumulation mediates the observed changes in cell adhesion molecule expression. Treatment with U18666A (5 μg/mL), a pharmacological inducer of intracellular cholesterol accumulation independent of ABCA1 inhibition, suppressed cell proliferation and induced morphological changes similar to those observed after lefamulin exposure (Figure 7I,J). Consistent with cholesterol accumulation, we observed cytoplasmic lipid droplet formation (Figure 7K) and increased mean fluorescence intensity (Figure 7L). Finally, RT-qPCR analysis was performed to evaluate changes in the expression of genes involved in cell adhesion molecule pathways following U18666A treatment. The mRNA levels of *Ptprc*, *H2-DMb1*, *Pecam1*, *Cldn4*, and *H2-B1* were upregulated, consistent with the transcriptional changes induced by lefamulin or tiamulin (Figure 7M), whereas *Itgb7* expression was not increased. Togethor, these findings indicate that pleuromutilin-induced intracellular cholesterol accumulation suppresses tumor cell proliferation and alters adhesion molecule expression, potentially contributing to immune microenvironment remodeling.

## 4. Discussion

Drug repurposing offers a time-efficient and cost-effective strategy for drug development [24]. Based on previous evidence suggesting the anticancer potential of pleuromutilin antibiotics [7,8,9], we hypothesized that lefamulin and tiamulin could serve as antitumor agents in HCC. In this study, we demonstrate that both agents inhibit HCC growth across cell lines, patient-derived organoids, and a mouse model. Although the dose used (37 mg/kg/day) exceeds typical antibacterial exposure [6], which may raise concerns about off-target effects or novel toxicities [25], our safety assessments in a normal hepatocyte line (THLE-2) and major organs did not reveal evident toxicity, suggesting a potential therapeutic window.

Mechanistically, lefamulin is known to inhibit bacterial protein synthesis by binding to peptidyl transferase center of the 50S ribosomal subunit [6]. However, transcriptomic analysis in tumor cells did not reveal significant enrichment of protein synthesis-related pathways. Instead, we observed consistent downregulation of *Abca1* and alterations in cell adhesion-related signaling pathways, suggesting that pleuromutilins may disrupt cholesterol homeostasis and tumor immune regulation. The lack of transcriptional changes associated with mitochondrial dysfunction further indicates that the observed antiproliferative effects are unlikely to result from non-specific cytotoxicity.

Metabolic dysregulation is a hallmark of cancer. In HCC, aberrant lipid metabolism contributes to tumor progression, making regulators such as SREBP1/2 and downstream effectors (e.g., SCD1, FASN, ACC, HMGCR) attractive therapeutic targets [26,27,28,29,30,31]. Furthermore, lipid metabolic disorders directly influence anti-tumor immunity, as cholesterol availability affects macrophage polarization and CD8^+^ T-cell function [32,33]. These observation highlight lipid metabolism as a dual-functional therapeutic axis affecting both tumor growth and immune response.

Excess intracellular cholesterol can induce lipotoxic stress and cell death when buffering mechanisms—such as esterification by ACAT1 (Acetyl-CoA Acetyltransferase 1) or efflux via ABCA1—are impaired [34,35,36]. ABCA1 exhibits context-dependent roles in cancer, promoting tumor progression in certain settings while acting as a tumor suppressor in others [37,38,39]. In our study, pleuromutilin treatment downregulated ABCA1 in Hepa1-6 cells, leading to intracellular cholesterol accumulation and aberrant distribution, consistent with previous reports linking cholesterol transport inhibition to endoplasmic reticulum stress-mediated cell death [40]. Although ABCA1 has been studied in the context of drug resistance, its direct contribution to tumor suppression and immune modulation remains incompletely defined. The absence of clinically approved ABCA1-targeting agents underscores a potential therapeutic gap, despite promising preclinical agents such as JNJ-26854165 [41]. We therefore propose that ABCA1 downregulation and subsequent cholesterol-induced toxicity represent key mechanisms underlying pleuromutilin-mediated tumor inhibition. Additionally, ABCA1 can suppress SREBP activity via negative feedback, potentially downregulating lipid synthesis and further constraining tumor growth [3,4,5,26,42]. This secondary metabolic disruption warrants further mechanistic investigation.

Immunotherapy has revolutionized HCC treatment through the use of immune checkpoint inhibitors, adoptive cell therapy, and cancer vaccines [5,43,44,45]. However, its efficacy is often limited by factors like inadequate T-cell infiltration and an immunosuppressive microenvironment [46], driving the need for combination strategies [47]. Cell adhesion molecules have emerged as important regulators of immune cell trafficking and activation [22,48,49,50]. Our RNA-Seq data reveal that pleuromutilin treatment alters the expression of multiple adhesion-related genes (*Ptprc*, *H2-DMb1*, *Itgb7*, *Pecam1*, *Cldn4*, *H2-Bl*, *Madcam1* and *Cntnap2*) involved in immune regulation [15,16,17,19]. We confirmed that this transcriptional shift has a functional impact, as treated tumors exhibited significantly increased CD8^+^ T-cell infiltration. This suggests that pleuromutilins can favorably modulate the tumor immune microenvironment, highlighting their potential for combination with immunotherapies.

The functional link between cholesterol metabolism and immune modulation may be mediated by lipid rafts—cholesterol-rich membrane microdomains critical for adhesion molecule recruitment and signaling [23,51,52,53]. We therefore propose a unifying mechanistic model in which pleuromutilin-induced ABCA1 downregulation leads to intracellular cholesterol accumulation, resulting in lipid raft remodeling and altered adhesion molecule signaling, thereby promoting cytotoxic T-cell recruitment.

Despite these promising findings, several limitations should be acknowledged. Transcriptomic enrichment analyses cannot exclude off-target effects, and phenocopy experiments using simvastatin, ABCA1 knockdown, and U18666A do not necessarily indicate identical molecular mechanisms. Further studies are required to delineate the precise links between cholesterol dysregulation, tumor cell proliferation, and immune signaling pathways. Nevertheless, our results highlight cholesterol homeostasis as a previously underappreciated therapeutic vulnerability in HCC.

## 5. Conclusions

Lefamulin and tiamulin inhibit HCC growth in vitro and in vivo by downregulating ABCA1, inducing intracellular cholesterol accumulation, and modulating cell adhesion-related signaling pathways. These effects are associated with enhanced CD8^+^ T-cell infiltration, suggesting a role in tumor immune microenvironment remodeling. Our findings identify pleuromutilin antibiotics as promising repurposed therapeutic candidates for HCC, with potential value in combination immunotherapy regimens.

## Data Availability

The raw data supporting the conclusions of this article will be made available by the authors on request.

## Supplementary Materials

The following supporting information can be downloaded at https://www.mdpi.com/article/10.3390/cancers18071054/s1: Figure S1. Neither lefamulin nor tiamulin affected the infiltration of CD4^+^ T cells or F4/80^+^ macrophages in vivo. Table S1. Primer sequences for RT-qPCR.

## Abbreviations

The following abbreviations are used in this manuscript:

ABCA1	ATP-binding cassette transporter A1
ACAT1	Acetyl-CoA Acetyltransferase 1
ACC	Acetyl-CoA carboxylase
Cldn4	claudin 4
Cntnap2	contactin-associated protein-like 2
DMSO	dimethyl sulfoxide
FASN	fatty acid synthase
GSEA	Gene Set Enrichment Analysis
H2-Bl	histocompatibility 2, blastocyst
H2-DMb1	histocompatibility 2, class II, locus Mb1
HCC	hepatocellular carcinoma
H&E	Hematoxylin and eosin
HMGCR	3-hydroxy-3-methylglutaryl-CoA reductase
IC_50_	half-maximal inhibitory concentration
IHC	immunohistochemical
Itgb7	integrin subunit beta 7
Madcam1	mucosal vascular addressin cell adhesion molecule 1
Pecam1	platelet endothelial cell adhesion molecule 1
Ptprc	protein tyrosine phosphatase receptor type C
RNA_Seq	RNA sequencing
SCD1	stearoyl-Coenzyme A desaturase 1
