# Supplementary material for: Pleuromutilins Suppress Hepatocellular Carcinoma Growth via ABCA1 Inhibition-Induced Cholesterol Accumulation
Source: Cancers (Basel). 2026 Mar 24;18(7):1054. doi: 10.3390/cancers18071054 (PMC13072156; doi:10.3390/cancers18071054)
Supplement: Supplementary file 1 [file cancers-18-01054-s001.zip › cancers-4156471-supplementary.pdf]

Supplementary Materials

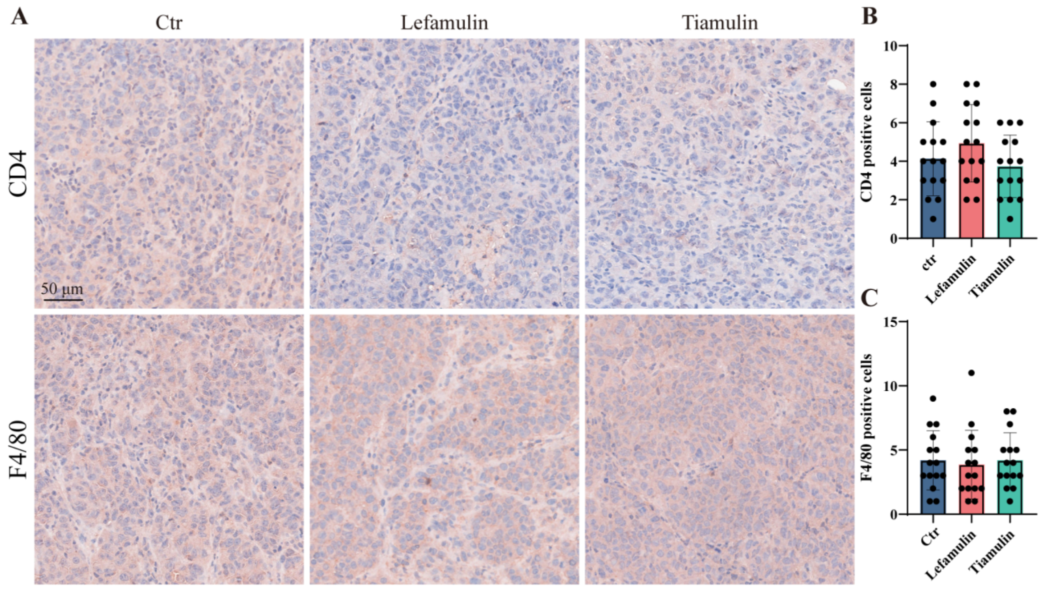

Figure S1: Neither lefamulin nor tiamulin alters the infiltration of CD4⁺ T-cells or F4/80⁺ macrophages *in vivo*. (**A**) Representative immunohistochemical images showing CD4⁺ T cells and F4/80⁺ macrophages in tumor tissues from mice treated with lefamulin or tiamulin (37 mg/kg/day) for 11 days. Scale bar, 50 μm. (**B**) Quantification of CD4⁺ T-cell infiltration (n=15). (**C**) Quantification of F4/80⁺ macrophage infiltration (n=15). Data are presented as mean ± SEM. Statistical analysis was performed using one-way ANOVA.

Table S1. Primer sequences for RT-qPCR

	Sequence (5'→3')
Ptprc_F	AGTGCATGTAGCTAGCAAGTGG
Ptprc_R	TGTAGGTGTTTGCCCTGTGAC
H2-DMb1_F	CTACCCGGAAGGACAGCAT
H2-DMb1_R	ACCTCATTTCAGCTCACCTCA
Itgb7_F	ATCACACCCGTGCCATCATA
Itgb7_R	GTAGAGAGGATTGTTGTCCTGC
Pecam1_F	GAGCCTCACCAAGAGAACGG
Pecam1_R	CCCAACATGAACAAGGCAGC
Cldn4_F	TGTAGAGTGGATGGACGGGT
Cldn4_R	CTCAGAGGGGCCAACTCAAG
H2-B1_F	GCAGCAGAAAAATAGCCATCCT
H2-B1_R	ACTCCACTAATCAACCCTCAGC
Madcam1_F	GAGATACAAGAGGCTGAGGGC
Madcam1_R	GAGGTCTGGCTCTGTAGTACTGG
Cntnap2_F	CGGTTCCCTTCAAGAACCTCA
Cntnap2_R	GGCTCATCACATTTTTGGAACG
